# Study protocol for a randomized control trial to investigate the effectiveness of an 8-week mindfulness-integrated cognitive behavior therapy (MiCBT) transdiagnostic group intervention for primary care patients

**DOI:** 10.1186/s12888-019-2411-1

**Published:** 2020-01-06

**Authors:** Sarah Frances, Frances Shawyer, Bruno Cayoun, Joanne Enticott, Graham Meadows

**Affiliations:** 1 Southern Synergy, Department of Psychiatry, School of Clinical Sciences at Monash Health, Monash University, Clayton, Victoria 3800 Australia; 2 Department of Psychiatry, School of Clinical Sciences at Monash Health, Monash University, Dandenong Hospital, 126 - 128 Cleeland St, Dandenong, Victoria 3175 Australia; 3 Mindfulness-integrated Cognitive Behavior Therapy Institute, Hobart, Tasmania Australia; 4 Department of General Practice, School of Primary and Allied Health Care, Faculty of Medicine, Nursing and Health Sciences, Monash University, Melbourne, VIC Australia; 5 Mental Health Program, Monash Health, Melbourne, Victoria Australia; 6 Melbourne School of Population and Global Health, University of Melbourne, Parkville, Victoria 3010 Australia

**Keywords:** Mindfulness-integrated cognitive behavior therapy, MiCBT, Mindfulness-based intervention, Mindfulness, Transdiagnostic, Anxiety, Depression, Equanimity, Group therapy

## Abstract

**Background:**

Effective transdiagnostic treatments for patients presenting with principal or comorbid symptoms of anxiety and depression enable more efficient provision of mental health care and may be particularly suitable for the varied population seen in primary healthcare settings. Mindfulness-integrated cognitive behavior therapy (MiCBT) is a transdiagnostic intervention that integrates aspects of CBT, including exposure skills targeting avoidance, with training in mindfulness meditation skills adopted from the Vipassana or insight tradition taught by the Burmese teachers U Ba Khin and Goenka. MiCBT is distinguished from both cognitive therapy and mindfulness-based cognitive therapy by the use of a theoretical framework which proposes that the locus of reinforcement of behavior is the interoceptive experience (body sensations) that co-arises with self-referential thinking. Consequently, MiCBT has a strong focus on body scanning to develop interoceptive awareness and equanimity. Designed for clinical purposes, the four-stage systemic approach of MiCBT, comprising intra-personal (Stage 1) exposure (Stage 2), interpersonal (Stage 3), and empathic (Stage 4) skillsets, is a distinguishing feature among other mindfulness-based interventions (MBIs). The aim of this study is to investigate whether and how group MiCBT decreases depression and anxiety symptoms for patients with a range of common mental health conditions.

**Methods:**

Participants (*n* = 120) recruited via medical practitioner referral will be randomized to MiCBT or a wait-list control. Inclusion criteria are age 18–75; fluent in English and having a Kessler Psychological Distress Scale (K10) score of 20 or more. The MiCBT treatment group receive an 8-week MiCBT intervention delivered in a private psychology practice. Participants complete a suite of online self-report measures and record the amount of meditation practice undertaken each week. The control group receive usual treatment and complete the measures at the same time points. Primary outcome measures are the Depression Anxiety Stress Scale-21 (DASS-21) and K10. Analysis will use mixed-model repeated measures.

**Discussion:**

The potential ability of MiCBT to provide a comprehensive therapeutic system that is applicable across diagnostic groups would make it an attractive addition to the available MBIs.

**Trial registration:**

This trial is registered with the Australia and New Zealand Clinical Trials Registry: ACTRN12617000061336; Date of registration: 11th January 2017.

## Background

General symptoms of anxiety and depression are commonly present across a range of psychological, somatic and medical conditions and contribute to a poorer medical prognosis. Developing effective and enduring transdiagnostic treatments for patients presenting with general symptoms of anxiety and depression, with or without concomitant physical illness, means that therapy can be efficiently provided to a broad range of clients in a variety of therapeutic settings and may be particularly suitable for application in the varied population seen in primary care settings.

### Cognitive behavior therapy

Since the 1970s, Cognitive Behavior Therapy (CBT) has enjoyed a dominant position in the plethora of available psychological therapies. It is one of the most widely used approaches and is regarded as treatment of choice for depression, either on its own or in conjunction with pharmacotherapy [1–3]. The efficacy of CBT has been shown across many diagnoses including depression, anxiety [4] and obsessive-compulsive disorder [5–7]. Data from meta-analyses also support the use of CBT for a range of disorders [8, 9]. The cognitive model used in CBT utilizes a structured experimental and collaborative approach to enable recognition and reappraisal of habitual dysfunctional and distorted thought patterns or behaviors [10].

### Mindfulness-based programs and interventions

The past three decades has witnessed the emergence of a so-called “third wave” [11] of cognitive and behavioral therapies, often referred to as mindfulness-based interventions (MBIs). These interventions place less importance on the reframing of thought content and behavior modification, and more emphasis on changing the relationship with thoughts in order to prevent their proliferation [12]. A shift towards disengaging or decentering from thought content is proposed to reduce the tendency for recurrence of depressogenic thoughts contributing to depressive relapse. More generally, MBIs encourage mindful acceptance of difficult psychological experience [13], taking a more holistic approach to psychological health that includes developing metacognitive awareness together with an accepting and compassionate attitude [2, 14]. The MBIs that will be discussed here are those that emphasize formal meditation practices similar to MiCBT in that they require formal meditation practices, namely mindfulness-based stress reduction (MBSR − [15]), developed as a stress management program for medical patients, and programs developed from MBSR, including mindfulness-based cognitive therapy (MBCT - [12, 16]), developed as a relapse prevention program for depression. While acceptance and commitment therapy and dialectical behavior therapy are considered to belong to the body of third wave behavioral therapies, they do not include formal mediation practice as a key intervention.

There is increasing evidence to support the clinical use of MBIs for both physical and psychological conditions, including chronic pain and psoriasis [15, 17–19], cancer [20, 21], diabetes [22], depression [23] substance abuse [24, 25] and anxiety [26]. Programs such as the mindfulness-based eating program (MB-EAT- [27, 28]), mindfulness-based relationships enhancement (MBRE − [29]) have been developed for specific issues and are based on the MBSR or MBCT protocols. In the United Kingdom, MBCT is now listed in the National Institute for Health and Care Excellence (NICE) guidelines as an evidence-based treatment for relapse prevention in depression [30]. The Agency for Healthcare Research and Quality conducted a comprehensive review of meditation programs and found moderate strength of evidence for the benefit of mindfulness meditation programs for stress and for pain severity, moderate to low strength of evidence that mindfulness meditation, as taught in MBSR-based MBIs, decreases symptoms of anxiety, depression, and perceived stress/general distress, and reports low effects for stress and mental health-related quality of life, positive mood, attention and weight [31].

### Mindfulness-integrated cognitive behavior therapy (MiCBT) - why another MBI?

MiCBT was developed over 15 years ago in Australia independently from MBSR and MBCT and has gained increasing recognition in recent years, especially following the publication of a protocol for an 8-week MiCBT program [32]. MiCBT was designed to be used as a transdiagnostic intervention that is firmly grounded in a CBT approach, which includes a structured week by week protocol, use of Socratic questioning, exposure techniques and behavioral experiments as well as daily mindfulness meditation practices.

In MiCBT, it is proposed that thoughts and body sensations co-emerge and that the type and hedonic tone of body sensations have a key role in determining the type and intensity of behavioral reactivity.

The mindfulness meditation skills that are taught in MiCBT enable participants to understand and recognize co-emergence mechanisms experientially, as explained below. Following the introduction of attention training with mindfulness of breath, a series of body scanning practices are taught to develop interoceptive awareness together with equanimity (non-reactivity) so that interoceptive signals are experienced and understood to be transient events. The ability to remain equanimous towards sensations has a powerful impact on neutralizing reactivity.

**Fig. 1:**
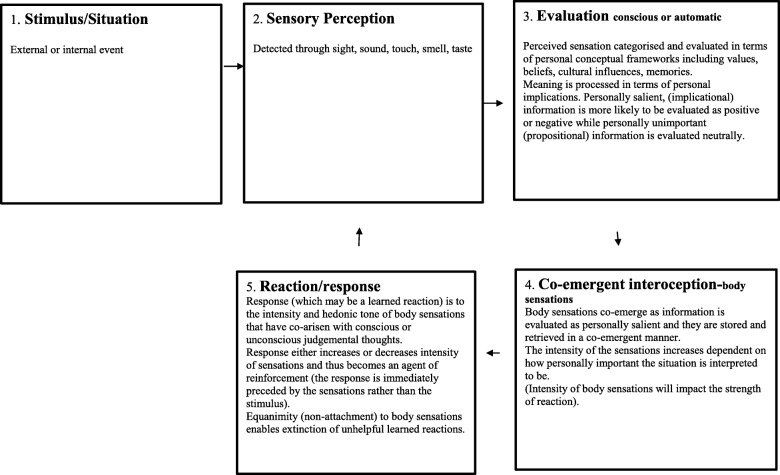
Co-emergence model

**Fig. 2:**
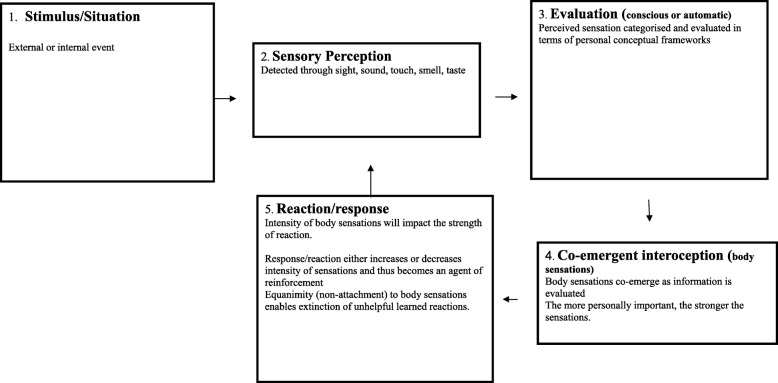
Co-emergence model in disequilibrium

The co-emergence model of reinforcement (see Fig. 1) proposes that a stimulus is perceived through the senses, (hearing, tasting, smelling, seeing, touching) and once perceived is then interpreted, evaluated and contextualized in terms of personal relevance. The model depicts that the stimulus may either be generated internally (as thoughts, memories or body sensations) or externally from the environment. The co-emergence model, in line with operant conditioning principles, proposes that when an experience is evaluated self-referentially (associated with “I”, “me” or “mine”), evaluative thoughts co-emerge with body sensations (interoception). The more the stimulus is evaluated as being personally relevant, the more intense body sensations are perceived to be. These sensations are experienced as pleasant, unpleasant or neither pleasant nor unpleasant, and this determines the desire for more or less of the experience, which then prescribes a reaction; attachment or avoidant behavior. When the reaction successfully increases pleasant or decreases unpleasant hedonic tone, it is reinforced so that the locus of reinforcement is proposed to be the interoceptive experience. The co-emergence model (in Fig. 1) is depicted as a system in equilibrium, in which all components receive adequate attention. The model proposes that in this balanced state, emotional disorders are not likely to occur. In psychopathology, however, the system is seen in a maintained state of disequilibrium (Fig. 2), with depleted attention in the sensory perception and interoception components and increased attention and over-processing in the evaluation and reaction components. The goal of MiCBT is to restore the system’s homeostatic balance by decreasing the amount of processing in over active components (evaluation and reaction) and reallocating attention to areas of depletion (sensory perception and co-emergent interoception), in situational, interpersonal and intra-personal contexts [32].

**Fig. 3:**
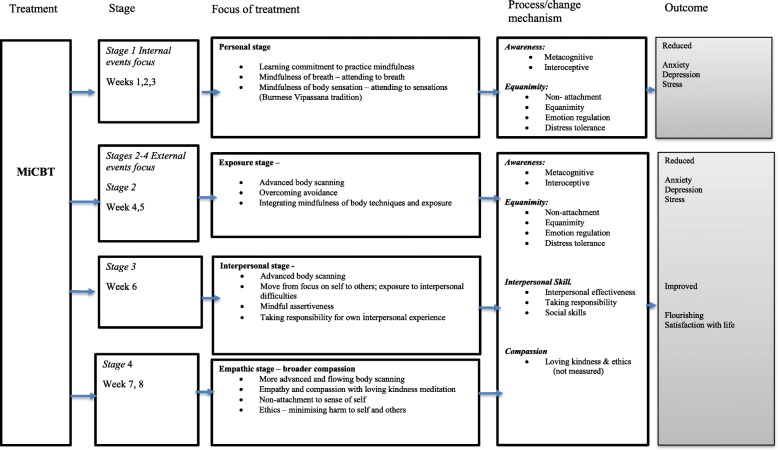
MiCBT Process model

The MiCBT program structure (depicted in Fig. 3) is designed to systematically develop skills to achieve this rebalancing process. Stage 1 (weeks 1–3) teaches mindfulness meditations, both breath meditation and body scanning to develop attention regulation, metacognitive and interoceptive awareness together with response inhibition or non-reactivity/equanimity. Stage 2 (weeks 4–5) further develops interoceptive awareness, teaching the first body scan in a series of advanced and systematic scanning methods to enable sensitivity to even very subtle body sensations in all areas of the body, followed by imaginal and in vivo exposure techniques for managing avoidance. Stage 3 (week 6) uses faster body scanning techniques and uses exposure methods to address interpersonal issues and assertiveness. Stage 4 (weeks 7–8) teaches deeper levels of body scanning and introduces awareness of ethics and compassion for self and others. Increased awareness of, and equanimity towards, body sensations facilitates the introduction of behavior change strategies so that imaginal and in-vivo exposure to avoided situations (including interpersonal situations) becomes manageable. This practical technique positively impacts social functioning, enabling difficult avoided situations and conversations to be possible. The resultant improved social functioning and connectedness with others tends to enhance well-being and flourishing [33] and assist in preventing relapse [34]. The explicit practice of compassion and personal ethics as an aspect of psychological well-being, combined with the use of systematic exposure techniques, are features distinguishing MiCBT from other MBIs.

In terms of the mindfulness meditation components of MiCBT, the predominance of body scanning exercises is important because anxiety and mood disorders have been associated with inability to appropriately perceive and manage the interoceptive cues associated with disturbing phenomena [35, 36]. Given the increasing evidence of the depleted interoceptive processing in mental health disorders [36], psychological disorders may be considered to involve disturbances of homeostatic and allostatic mechanisms creating reduced ability to respond effectively to the present moment and maintaining learned reactivity, as opposed to responsiveness to current interoceptive signals [37]. From this perspective, anxiety and depression could be conceptualized as interoceptive disorders, in which homeostasis is habitually disrupted through self-referential interpretations of events, creating changes to interoceptive states. Thus, anxiety and depression manifest disturbed interoceptive awareness and compensatory top-down processing [38].

The rationale for learning to develop high levels of interoceptive awareness through body scanning is consistent with findings that interoceptive awareness (IA) is a pre-requisite for effective emotion regulation [39]. As described earlier, MiCBT rests on the proposition that the extent to which thoughts are interpreted as personally implicational reflects in the intensity of the physiological arousal (experienced as body sensations), which in turn determines the resultant behavior [40]. This resultant behavior may be a rationally thought out response or a learned and automatic reaction. Accordingly, the ability to be equanimous to arising body sensations downregulates emotional reactivity [41] and the consequent “dis-ease” that characterizes many psychological disorders.

Both MiCBT and MBCT clearly have shared origins in Buddhist psychology and teach focused attention, awareness and decentering. MBCT was founded on a cognitive model of depressive relapse and the skills taught, particularly awareness and decentering, aim to help patients recognize and step out of the automatic patterns of negative thinking that underlie depressive relapse [42]. The focus in MBCT is on depressive relapse prevention because the “risk of relapse and recurrence is brought about by increased cognitive reactivity to small changes in depressed mood” [43].

MiCBT is differentiated from MBCT rather in its operationalization of techniques than in its Buddhist philosophical underpinnings. A notable difference in MiCBT is its particular focus on the somatic, as well as the cognitive aspect of internal experience such that sensations co-arising with thoughts (at the conscious or sub-conscious level of awareness) are posited to be the locus of behavioral reinforcement. Thus, MiCBT has a cogent rationale for its applicability across clinical diagnoses because the underlying causes of mental disturbance are posited to be reactivity to interoceptive signals (co-arising with thoughts) that are deemed to be unacceptable. Therefore, MiCBT potentially offers a distinctive contribution amongst the suite of mindfulness interventions now available.

Interestingly, despite the inclusion of body-based activities to develop body awareness, including yoga exercises and mindful walking in programs like MBSR and MBCT, many mindfulness scales used in researching these programs make minimal reference to interoceptive awareness. The current research has attempted to address this issue by the inclusion of a measure of interoceptive awareness, the *Multidimensional Assessment of Interoceptive Awareness* (MAIA -[42, 44]).

### MiCBT existing evidence

The publication of the book *Mindfulness Integrated CBT: Principles and Practice* [32] has enabled a manualised protocol for the delivery of MiCBT to be in the public domain. There is a growing number of clinicians trained in MiCBT and utilising this therapy with a range of clients, including children [32], but the increasing empirical evidence for the efficacy of MiCBT remains limited. Besides transdiagnostic group applications reported by non-controlled pilot trials [45],there are RCTs supporting the efficacy of MiCBT in a variety of contexts: drug and alcohol setting (Wickham K: The effect of Mindfulness-integrated Cognitive Behaviour Therapy (MiCBT) on the experience of addiction, unpublished), depression with diabetes [46], procrastination and perfectionism in students [47], anxiety and depression in multiple sclerosis patients [48]; anxiety and depression in pregnant women [49], chronic pain [50], and sports anxiety and pessimism [51], suggesting that the delivery of the MiCBT treatment program in relatively homogenous group therapy settings shows good results across different patient populations. However, the claim that MiCBT is effective as a transdiagnostic group approach has not been demonstrated in heterogenous groups through a rigorous randomized controlled trial to date.

### Aims and hypotheses

The primary aim of this study is to evaluate the effectiveness of a group delivery of MiCBT across a range of psychological disorders in a private practice setting. The secondary aim is to examine mechanisms of action underpinning MiCBT. It is hoped that the findings will also add to the body of knowledge on outcome measurement with mindfulness-based approaches, in particular the role of interoceptive, metacognitive awareness and equanimity as key mechanisms of change. Based on a preliminary pilot study [52] and experience of colleagues using MiCBT clinically, we hypothesize that the transdiagnostic application of MiCBT implemented in group format in a private psychology clinic will be more beneficial than the treatment-as-usual provided in the wait-list group. The hypotheses are that, compared to the wait-list control group, the MiCBT group will show greater decrease in clinical symptoms (self-reported levels of depression, anxiety and stress, life satisfaction and flourishing). It is further hypothesized that these improvements will be maintained over a six-month period post intervention. The study will also examine the key mechanisms of action. It is hypothesized that 1) improvements in depression, anxiety and stress scores as a function of MiCBT relative to the control group will be mediated by a) improvements in awareness (metacognitive and interoceptive awareness) and b) equanimity. Given the contribution of positive relationships with others to well-being and flourishing noted earlier, we were also interested in exploring the degree to which 2) changes in life satisfaction and flourishing scores are mediated by improvements in interpersonal skills compared to awareness and equanimity.

## Methods/design

This study is a randomized controlled trial as it is an early stage trial. Participants will be recruited through medical practitioner referral from the metropolitan region of Melbourne randomized to one of two treatment conditions: MiCBT or a control condition. Both conditions will continue with usual treatment which may include medication and/or ongoing psychological therapy throughout the trial. It was not feasible due to lack of resources to conduct an active control for this study which will be conducted in a private practice setting in the context of a PhD project. However, a wait-list control condition control is appropriate for such an early phase RCT. In addition, the question as to whether any positive treatment outcomes reflect specific or general therapy effects is being addressed to some extent through the mediation analyses.

### Eligibility criteria

Inclusion criteria are age 18–75; fluent in English and with a K10 score of 20 or more. Exclusion factors include: less than 18 years of age, non-English speakers, current psychotic symptoms, current diagnosis of borderline or antisocial personality disorder, and current drug or alcohol dependency, pervasive developmental delay, organic mental disorder, prescribed more than 20 mg diazepam equivalent per day. Informed consent is obtained by the researcher from all participants prior to commencement of the program.

### Intervention

The MiCBT intervention is adapted from the published protocol [32] with the variation that individual sessions between each group session are not be routinely offered; such sessions are only offered to a participant if a crisis emerges that is deemed too complex to address in a group situation. The MiCBT group will receive the MiCBT treatment in a group format comprising weekly two-hour sessions to groups of 10–15 participants for 8 weeks. The program is delivered by one MiCBT-trained psychologist with 9 years of experience in delivering MiCBT, including twenty-five MiCBT groups. The intervention is administered in a psychology clinic. Participants will be asked to engage in specific mindfulness meditation for an hour each day; two half hour practice sessions and are asked to log their meditation practice hours. Audio instructions for the mindfulness exercises for each session are provided from Cayoun’s (2015) text (used with permission). At the start of each session there is a facilitated group meditation for half an hour which introduces the meditation practice for the following week. There is a review and inquiry process about the experiences of the previous weeks practice and in the second hour of each session there is a psycho-educational component explaining the rationale for the following weeks practice or introducing specific homework designed to apply the skills acquired (e.g. an exposure task to avoided behaviors). Attendance at each session is recorded and non-attenders are followed up. Figure 3 maps the putative processes and outcomes in MiCBT.

### Participant timeline

The baseline measure is the K10 administered by the General Medical Practitioner as part of the referral process. The remaining assessments will be administered 1 week prior to the commencement of the program post randomization (see Table 1) in order to fully standardize the assessment time frame across participants and address the considerable and varied delay between recruitment and commencing the program. Administering pre-treatment assessments as close as possible to the start of the intervention is expected to have the advantage of achieving maximum precision and sensitivity to change in the context of fluctuating mental health conditions with only low risk of systematic observer or participant bias which could nevertheless be readily gauged though pre- and post-randomization K10 analysis. Observer bias is unlikely because the assessments are conducted on-line. Participant bias, such as demoralization following allocation to control, is also considered unlikely as all participants will receive the program within a relatively short time frame and participants who express a strong desire to be allocated to MiCBT immediately will be recommended to seek alternative treatments and alternative treatments are recommended for participants who express a strong desire to be allocated to MiCBT immediately.

**Table 1:**
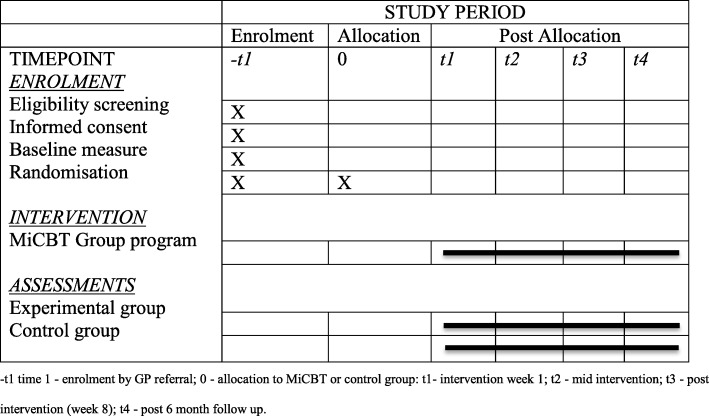
Timeline for enrolment, allocation, intervention and assessment. -t1 time 1 - enrolment by GP referral; 0 - allocation to MiCBT or control group: t1- intervention week 1; t2 - mid intervention; t3 - post intervention (week 8); t4 - post 6 month follow up

### Measures

The intent of this study is to examine the effectiveness of MiCBT to create changes in clinical measures of depression, anxiety and stress. It is hypothesized that these changes will occur during the program in stages 1,2 and 3 and be enhanced in stage 4 because of the additional practice time. Compassion and ethics are taught in Stage 4 for relapse prevention which is not the focus of the current study.

#### Kessler Psychological Distress Scale (K10)

The K10 is a widely-used 10-question screening scale of psychological distress that focuses on anxiety and depressive symptoms experienced in the last 4 weeks. It has been shown to have good psychometric properties across major sociodemographic groups. The scales show excellent internal reliability and strongly discriminate between clinically significant disorders as defined in the DSM-IV and non-clinically significant disorders [53]. This measure was chosen because it is most commonly used by general practitioners in Victoria as a screening tool to identify those eligible for a Mental Health Care Plan and subsidized psychological treatment.

#### Depression, Anxiety, and Stress Scale (DASS-21)

The DASS-21 is a 21-item version of the DASS-42 and is a self-report questionnaire designed to measure three domains: depression, anxiety and stress [54] [55, 56]. Each subscale contains 7 items, and items are rated on a four-point likert scale. Higher scores indicate more severe symptoms. The DASS-21 is reported to have high internal consistency for each of the subscales (depression, r = .88; anxiety, r = .82; stress r = .90), and the total scale (r = .93) [55, 56].

#### Satisfaction with Life Scale (SWLS)

This is a widely used 5-item self-report measure of life satisfaction. The scale is conceptualized as having two components, a cognitive judgmental component and an affective or emotional component [57]. The SWLS uses a 7-point Likert scale from 1 (*strongly disagree)* to 7 (*strongly agree*). The range of possible scores is from minimal satisfaction with life (5) to very high satisfaction with life (35). The SWLS has been shown to have convergent validity with other self-report measures of life satisfaction, including the Philadelphia Geriatric Center Morale Scale [58]. The SWLS appears to tap a single life satisfaction factor. Internal consistency is good with a Cronbach’s alpha coefficient of .83.

#### The Flourishing Scale (FS)

The FS is an eight item self-report measure of psychological well-being [59]. The scale taps into aspects of human functioning such as feelings of competence, positive relationships, and purpose in life. It uses a 7-point Likert scale with responses from strong disagreement to strong agreement. Scores range from 8 to 56 and high scores indicate positive self-appraisal of psychological well-being. The scale developers report strong correlations with other psychological well-being scales and good internal consistency with Cronbach’s alpha coefficient of 0.87.

#### Mindfulness-based Self-Efficacy Scale-Revised (MSES–R)

This 22-item self-report measure is designed to assess the changes in levels of perceived self-efficacy before, during and after mindfulness-based therapy [32]. There are six subscales, Emotion Regulation, Distress Tolerance, Equanimity (considered here as measuring aspects of equanimity), Taking Responsibility, Social Skills, and Interpersonal Effectiveness (considered here as measuring aspects of interpersonal skills). Items are rated on a 5-point Likert scale from 0 (*not at all*) to 4 (*completely*). Higher scores indicate higher mindfulness based self-efficacy. Subscales can be used separately to assist in identifying relative strengths and weaknesses or combined to provide a global score. The MSES-R total shows high internal consistency (Cronbach’s alpha .86) [32]; has a good divergent validity, inverse relationships with the DASS-21 [60], and good concurrent validity with the Freiberg Mindfulness Inventory (FMI) [61], the Mindfulness Attention and Awareness Scale (MAAS), [62], the Kentucky Inventory of Mindfulness (KIMS), [63, 64] and the Five Factor Mindfulness Questionnaire (FFMQ), [65, 66].

#### The Experiences Questionnaire (EQ)

This is a 20-item self-report measure of decentring [67] and is conceptualized as a protective factor and capable of measuring resilience to depressive relapse. Decentring is a key element of metacognitive awareness [68, 69] as reflected here: “We refer to the process of experiencing negative thoughts and feelings within a decentred perspective as metacognitive awareness..” [70]. The EQ uses a 5-point Likert scale with responses from “never” to “all the time”. This is a relatively new measure whose psychometric properties are still being investigated. A Spanish study [71] found (reliability: Cronbach’s α = .89; convergent validity: r > .46; and divergent validity: r < − .35).

#### Multi-dimensional Assessment of Interoceptive Awareness (MAIA)

This is a 32-item self-report measure of body awareness, both interoceptive and proprioceptive [44]. It uses a 6-point Likert scale with responses from 0 (never) to 5 (always). There are eight subscales; Noticing, Not-Distracting, Not-Worrying, Attention Regulation, Emotional Awareness, Self-Regulation, Body Awareness and Trusting [44]. The internal-consistency reliability of each of the 21 validity measures was reported by the scale developers as ranging from .70 to .93 (median .85) [44, 72].

#### The Nonattachment Scale (NAS)

This unidimensional measure is designed to tap the construct of being equanimous, flexible and receptive [73]. This appears to be congruent with cognitive flexibility, an attribute that is developed by mindfulness practice. The NAS is shown to correlate with a quality of consciousness related to constructs such as emotion regulation, interpersonal effectiveness, well-being and mental health [73]. It is a 30-item self-report scale and uses a 6-point Likert scale ranging from 1 (disagree strongly) to 6 (agree strongly). Internal consistency levels are >.80 (Cronbach’s alpha).

#### Meditation practice

The amount of meditation practice completed by each participant is recorded daily by each participant and then collected each week on an individualized form. They are not disclosed to other participants.

#### Adverse effects

There is a weekly check in with participants about their practice and any issues raised are addressed. In the event of participants experiencing difficulties in relation to their meditation practice the following actions may be taken: a) modify the type of practice for the coming week or b) provide some individual counselling to the participant. The 6-month follow-up questionnaire includes a question about the occurrence of any adverse effects or other difficulties thought to be as a result of meditating. The questions were taken from a recent study about the broad ranges of experiences that occur in association with meditation practices [74]. The questions are:

1. “Did you have any unexpected, challenging, or difficult experiences that you associate with your practice of meditation?”
2. “How did these experiences impact your life”? [74].

All measures will be administered on line using Qualtrics survey software, with the exception of the amount of meditation practice which will be recorded by each participant and reported privately on a personalized form each week. There are inadequate resources available for this PhD study to administer the qualitative questions in person.

Participants are emailed a link to the measures utlizing their unique identification number. Reminder emails are sent to those who do not respond and if there is still no response the participant is telephoned.

### Primary and secondary outcomes

The primary outcome measure is the DASS-21. The secondary outcome measures are the K10, SWLS and FS psychological well-being scales. Measurement will occur at baseline, mid-intervention, post intervention and at 6-month follow up.

### Mediator hypotheses

It is hypothesized that the mediators of the clinical change will be reflected by changes in equanimity (NAS), decentering (EQ), interoceptive awareness (MAIA) and Mindfulness-based self-efficacy (MSES-R). The following mediation hypotheses will be tested: 1) improvements in the DASS-21 and K10 (clinical symptoms) at the end of MiCBT treatment will be mediated by improvements in the MSES-R (equanimity-related subscales), EQ and NAS mid-intervention; 2) improvements in clinical symptoms (DASS-21 and K10) at the six-month follow-up will be mediated by improvements in the MSES-R (equanimity-related subscales), EQ and NAS at the end of treatment. We will also explore the degree to which 3) improvements in the SWLS and FS at the six-month follow up is mediated by improvements in the MSES-R (interpersonal skills and social skills subscales) compared to MSES (equanimity subscales), EQ and MAIA measured at the end of treatment.

Measurements of ethical awareness and compassion as addressed in stage 4 of the program were not included as they would make the number of items unmanageable but could be considered in future studies and their target is relapse prevention which not the focus of this study.

### Sample size

The trial is powered to detect significant differences relevant to the primary outcome. A power analysis (G-power 3.1) for medium effect (Cohen’s d = .5) was conducted with power of .08, and using the conventional significance level (.05), resulting in a desired sample of 102 (51 participants in each group). Consistent with previous studies (e.g. [75]) allowing for a conservatively estimated attrition rate of 20% to follow up, a sample of 120 will be recruited. Based on the projected sample size of 102, power of 0.8, and zero, small, medium or large effect sizes conditions for the indirect effect, the mediation analyses are well-powered to detect a medium beta paths between the independent variable and the mediator and between the mediator and the dependent variable (β = .0.39) using bootstrapping and the Sobel test [76]. Data will be collected using online self-report measures with the Qualtrics platform at the four measurement points; baseline, mid-intervention, post-intervention, and 6-months follow-up.

### Recruitment

General Practitioners and psychiatrists practicing in the local area will be mailed recruitment letters and information flyers about the study together with referral forms and an explanation of the project eligibility criteria all of which are approved by the Monash University Human Research Ethics Committee. Referring medical practitioners are requested to provide a K10 score for each potential participant to determine eligibility as part of their referral.

Mail-outs to medical practices will be repeated to maintain the flow of participants to meet the required numbers. Applicants are subsequently screened at initial face-to-face or telephone interview by the researcher. Participants are then provided with an explanatory statement describing the project and after reading it through, those who wish to participate in the study are invited to sign the consent to participate form.

### Randomization

All subjects will be allocated an identification number and then randomly allocated to either the MiCBT group or the control group. Randomization is stratified based on the K10 scores (Levels of psychological distress: mild and moderate K10 score < 30; severe, K10 score ≥ 30), antidepressant or mood stabilizer medication (yes/no) and gender. The randomization procedure is conducted using Stata Version 14 SE by a researcher independent from all other aspects of project implementation, including group facilitation and data collection, using random allocation of treatments balanced in blocks. A sequence of treatments (test or control) are randomly permuted in blocks of size 4. Blocks of size 4 were chosen to ensure a balance of treatments in all group sessions, because groups of size 8 could begin as soon as 16 participants are recruited. Allocation is stratified by three stratification variables, each having two values: gender (M/F), antidepressants (Y/N), K10 score (20+/30+). Therefore a randomization schedule is generated for each of the 8 strata: F N 20+; F N 30+; F Y 20+; F Y 30 + M N 20+; M N 30+; M Y 20+; M Y 30+.

### Blindness

With the exception of meditation practice data, which is collected as described, all other assessments are conducted online, thus minimizing the possibility of researcher bias. Analysis of results will not be blinded.

### Data collection

In addition to the quantitative measures previously described participants in the MiCBT group will be asked some additional qualitative questions at the end of the 8-week program evaluating the program and asking about any adverse events they may experience with their meditation practices during the MiCBT program. Additionally, at the 6-month follow up, participants in the MiCBT group will be asked questions relating to adherence to meditation practices and any changes to treatment. Participants in the control condition will complete all measures at the same four time points as the treatment group and receive treatment as usual during this time.

### Data management

Results of the assessments will be kept confidential. Participants are allocated a unique identifier to be used when processing and communicating results. Personal details and results will be kept in separate databases and stored in accordance with Australian Psychological Society Ethical Standards and as outlined in the approved ethics application. Any publication of the results will not be published in a manner that would reveal the identity of any participants. The research dataset, de-identified, may be made publicly available.

### Research ethics approval

The project has approval from Monash University Human Research Ethics Committee (Approval Number CF16/2278–2,016,001,131). This ethics approval addresses issues of confidentiality as described above, consent of participants, access to data, ancillary and post-trial care and limitations of dissemination to thesis transcript, journal articles/book chapters, conference papers.

### Statistical analysis

Statistical analysis will be conducted using SPSS version 24 statistical software package. The dependent variables are scores on the two primary outcomes (DASS-21 and the K10), the secondary outcomes (SWLS and FS) and the other mediators (EQ, the MSES-R, the MAIA, and the NAS). The expected outcomes are that the MiCBT group will show significantly changed scores as compared to the wait-list control group, with lower scores on the DASS-21, the K10 and higher scores on the SWLS, FS, the MSES-R, the MAIA, the EQ, and the NAS.

The differential changes in the MiCBT group compared to the control group is the primary focus of the analysis. Analysis will use mixed-model repeated measures (MMRM), the method recommended for clinical trial data [77]. Evidence for the effectiveness of MiCBT will be demonstrated by a significant two-way interaction demonstrating greater change in the outcome measures in the MiCBT group from the control group pre to post intervention. Baseline to follow-up interactions for the MiCBT group will also be examined and changes compared with post intervention data. Results of the trial will be made available to referring clinicians and to participants in the form of a summarized report. Results may be published in journal articles, student thesis and conference presentations.

Intention-to-treat (ITT) analysis will be used because this approach uses all available information. Subjects with incomplete data are not discarded and missing data are not replaced with estimated values. Missing data will be examined and if found to be missing completely at random (MCAR) then the analysis will proceed without replacing missing data with estimated values. If pattern of missingness is not MCAR then multiple imputation will be applied using the strata that defined the non-random missingness.

This is consistent with the CONSORT standard [78]. If data are non-normal, then it will be analyzed using generalized linear MMRM. The mediational impact of pre to post MAIA, MSES, EQ and NAS will be assessed using the Sobel test, a method for measuring indirect effects [79]. The test assesses the significance of the product of the coefficients for treatment-mediator and mediator-outcome effects. A bootstrapped multivariate extension of the Sobel test will be used to examine both the total indirect effect and the individual effect and the effect of each mediator. The first model will comprise: group (MiCBT vs wait-list control) as the independent variable; clinical DASS-21 and K10 measures as the dependent variables; and MAIA (interoceptive awareness), EQ (metacognitive awareness), MSES-R (Emotion Regulation, Distress Tolerance, Equanimity subscales and total) and NAS (equanimity), scales that reveal significant group x time interactions, as mediators. Two time-frames will be tested for changes over the course of the program itself (mediators measured at 4 weeks; outcomes at 8 weeks) and in the longer term (mediators measured at 8 weeks, outcomes at 6 months). The second model will comprise: group (MiCBT vs wait-list control) as the independent variable: psychological wellbeing SWLS and FS measures (measured at 6 months) as the dependent variables; and MSES-R subscales of Taking Responsibility, Social Skills, Interpersonal Effectiveness and total score (measured at 8 weeks), MAIA (interoceptive awareness), EQ (metacognitive awareness), MSES-R (Emotion Regulation, Distress Tolerance, Equanimity subscales and total) and NAS (equanimity) scales that reveal significant group x time interactions, as mediators.

## Discussion

MiCBT is a potentially important addition to the suite of mindfulness-based interventions because of its transdiagnostic applicability and the comprehensive nature of the intervention that seeks to directly address fundamental processes underpinning suffering, described by the co-emergent model of reinforcement. It employs a sophisticated integration of traditional mindfulness meditations including loving kindness, cognitive behavioral techniques as well as its consideration of personal ethics as an aspect of psychological well-being. This study aims to investigate the effectiveness of MiCBT as a group intervention with a transdiagnostic population. It will explore if participants who engaged in the MiCBT program show improvement in anxiety, depression, stress, well-being and flourishing compared to a treatment as usual control group. The study will also explore some of the putative underlying mechanisms of action, notably interoceptive and metacognitive awareness and equanimity, but also interpersonal effectiveness.

Study participants represent the population of mild to moderately psychologically unwell individuals whose mental health has considerable impact on their flourishing. This contributes to negative impacts on family well-being and community costs in terms of lost work days and costs associated with medical treatments [80]. The availability of treatment options is frequently limited with long wait times to join the MBIs that are offered through community mental health services while patients continue to consume valuable medical and psychiatric resources. The ability of MiCBT to be delivered trans-diagnostically means that it is much easier to recruit groups of a viable size without having a long waiting period. Delivery through private psychology practices utilizes the well-established therapeutic collaboration in Australia between general medical practitioners and psychologists in private practice, which is supported by the National Health Insurance Scheme (Medicare). While the intervention is of short duration, it comprehensively teaches specific self-regulation skills, both in personal and interpersonal contexts. Its short duration enables the program to be cost-efficient, and in Australia, to be delivered within the limits of government-subsidized group psychological treatments.

The datasets used and analyzed during the current study will be available from the corresponding author on reasonable request.

## Data Availability

The individual deidentified participant datasets generated and/or analysed during the current study will be made available in Monash University’s institutional data repository, Monash.figshare (https://monash.figshare.com/).

## Abbreviations

CBT: Cognitive Behavior Therapy
DASS 21: Depression, Anxiety and Stress Scale
EQ: Experiences Questionnaire
FS: Flourishing scale
ITT: Intention to treat
K10: Kessler Psychological Distress Scale
MAIS: Multidimensional Assessment of Interoceptive Awareness
MB-EAT: Mindfulness-based Eating Program
MBI: Mindfulness-based Intervention
MBRE: Mindfulness-based Relationship Enhancement
MBSR: Mindfulness-based Stress Reducyion
MiCBT: Mindfulness integrated Cognitive Behavior Therapy
MMRM: Mixed Model Repeated Measures
MSES: Mindfulness-based Self-efficacy Scale
NAS: Non-attachment Scale
NICE: National Institute for Health and Care Excellence
SWLS: Satisfaction with Life Scale

## References

[1] Bhar SS, Gelfand LA, Schmid SP, Gallop R, DeRubeis RJ, Hollon SD, Amsterdam JD, Shelton RC, Beck AT (2008). Sequence of improvement in depressive symptoms across cognitive therapy and pharmacotherapy. J Affect Disord.

[2] Churchill R, Moore TH, Davies P, Caldwell D, Jones H, Lewis G, Hunot V. Mindfulness-based'third wave'cognitive and behavioural therapies versus treatment as usual for depression. Cochrane Libr. 2010, Issue 9. Art. No.: CD008705. 10.1002/14651858.CD008705. 10.1002/14651858.CD008705 PMC4110888 25067907

[3] DeRubeis RJ, Hollon SD, Amsterdam JD (2005). Cognitive therapy vs medications in the treatment of moderate to severe depression. Arch Gen Psychiatry.

[4] Olatunji BO, Cisler JM, Deacon BJ (2010). Efficacy of cognitive behavioral therapy for anxiety disorders: a review of meta-analytic findings. Psychiatr Clin N Am.

[5] Barrett P, Farrell L, Dadds M, Boulter N (2005). Cognitive-behavioral family treatment of childhood obsessive-compulsive disorder: long-term follow-up and predictors of outcome. J Am Acad Child Adolesc Psychiatry.

[6] Park HS, SHIN YW, Ha TH, Shin MS, Kim YY, Lee YH, Kwon JS (2006). Effect of cognitive training focusing on organizational strategies in patients with obsessive-compulsive disorder. Psychiatry Clin Neurosci.

[7] Siev J, Chambless DL. Specificity of treatment effects: cognitive therapy and relaxation for generalized anxiety and panic disorders. Journal of consulting and clinical psychology. 2007;75(4):513. 10.1037/0022-006X.75.4.513 17663606

[8] Butler AC, Chapman JE, Forman EM, Beck AT (2006). The empirical status of cognitive-behavioral therapy: a review of meta-analyses. Clin Psychol Rev.

[9] Hofmann SG, Sawyer AT, Witt AA, Oh D (2010). The effect of mindfulness-based therapy on anxiety and depression: a meta-analytic review. J Consult Clin Psychol.

[10] Sacco WP, Beck AT (1995). Cognitive theory and therapy.

[11] Hayes SC, Strosahl KD, Wilson KG. Acceptance and commitment therapy, second edition: the process and practice of mindful change. New York: Guilford Publications; 2011.

[12] Segal Z, Williams J, Teasdale J (2002). Mindfulness-based cognitive therapy for depression: a new approach to relapse prevention.

[13] Meadows G, Singh B, Grigg M (2007). Mental health in Australia : collaborative community practice.

[14] Kahl KG, Winter L, Schweiger U (2012). The third wave of cognitive behavioural therapies: what is new and what is effective?. Curr Opin Psychiatry.

[15] Kabat-Zinn J, Wheeler E, Light T, Skillings A, Scharf M, J. Cropley T, Hosmer G, Bernhard JD (1998). Influence of a mindfulness meditation-based stress reduction intervention on rates of skin clearing in patients with moderate to severe psoriasis undergoing photo therapy (UVB) and photochemotherapy (PUVA). Psychosom Med.

[16] Segal ZV, Williams JMG, Teasdale JD (2013). Mindfulenss-based cognitive therapy for depression.

[17] Kabat-Zinn J, Lipworth L, Burney R (1985). The clinical use of mindfulness meditation for the self-regulation of chronic pain. J Behav Med.

[18] Kabat-Zinn J, Lipworth L, Burney R, Sellers W (1986). Four year follow-up of a meditation-based program for the self-regulation of chronic pain: treatment outcomes and compliance. Clin J Pain.

[19] Kabat-Zinn J (1982). An outpatient program in behavioral medicine for chronic pain patients based on the practice of mindfulness meditation: theoretical considerations and preliminary results. Gen Hosp Psychiatry.

[20] Campbell TS, Labelle LE, Bacon SL, Faris P, Carlson LE (2012). Impact of mindfulness-based stress reduction (MBSR) on attention, rumination and resting blood pressure in women with cancer: a waitlist-controlled study. J Behav Med.

[21] Speca M, Carlson LE, Goodey E (2000). A randomized, wait-list controlled clinical trial: the effect of a mindfulness meditation-based stress reduction program on mood and symptoms of stress in cancer outpatients. Psychosom Med.

[22] van Son J, Nyklíček I, Pop VJ, Pouwer F (2011). Testing the effectiveness of a mindfulness-based intervention to reduce emotional distress in outpatients with diabetes (DiaMind): design of a randomized controlled trial. BMC Public Health.

[23] Lenz AS, Hall J, Bailey Smith L (2016). Meta-analysis of group mindfulness-based cognitive therapy for decreasing symptoms of acute depression. J Spec Group Work.

[24] Cheisa A, Malinowski P (2011). Mindfulness-based approaches: are they all the same?. J Clin Psychol.

[25] Alfonso JP, Caracuel A, Delgado-Pastor LC, Verdejo-García A (2011). Combined goal management training and mindfulness meditation improve executive functions and decision-making performance in abstinent polysubstance abusers. Drug Alcohol Depend.

[26] Roemer L, Lee JK, Salters-Pedneault K, Erisman SM, Orsillo SM, Mennin DS (2009). Mindfulness and emotion regulation difficulties in generalized anxiety disorder: preliminary evidence for independent and overlapping contributions. Behav Ther.

[27] Kristeller JL (2003). Mindfulness, wisdom and eating: applying a multi-domain model of meditation effects. J Construct Human Sci.

[28] Kristeller JL, Hallett CB (1999). An exploratory study of a meditation-based intervention for binge eating disorder. J Health Psychol.

[29] Carson JW, Carson KM, Gill KM, Baucom DH (2004). Mindfulness-based relationship enhancement. Behav Ther.

[30] Abraham C, Kelly MP, West R, Michie S (2009). The UK National Institute for health and clinical excellence public health guidance on behaviour change: a brief introduction. Psychol Health Med.

[31] Goyal M, Singh S, Sibinga EM, Gould NF, Rowland-Seymour A, Sharma R, Berger Z, Sleicher D, Maron DD, Shihab HM (2014). Meditation programs for psychological stress and well-being: a systematic review and meta-analysis. JAMA Intern Med.

[32] Cayoun BA. Mindfulness-integrated CBT: principles and practice. New York: Wiley; 2011.

[33] Eraslan-Capan B (2016). Social connectedness and flourishing: the mediating role of hopelessness. Univ J Educ Res.

[34] Saeri AK, Cruwys T, Barlow FK, Stronge S, Sibley CG (2018). Social connectedness improves public mental health: investigating bidirectional relationships in the New Zealand attitudes and values survey. Aust & New Zealand J Psych.

[35] Pollatos O, Traut-Mattausch E, Schroeder H, Schandry R (2007). Interoceptive awareness mediates the relationship between anxiety and the intensity of unpleasant feelings. J Anxiety Disord.

[36] Khalsa SS, Adolphs R, Cameron OG, Critchley HD, Davenport PW, Feinstein JS, Feusner JD, Garfinkel SN, Lane RD, Mehling WE (2017). Interoception and mental health: a roadmap. Biological Psychiatry*:* Cognitive Neuroscience and Neuroimaging.

[37] Sterling P (2014). Homeostasis vs allostasis: implications for brain function and mental disorders. JAMA Psychiat.

[38] Paulus MP, Stein MB (2010). Interoception in anxiety and depression. Brain Struct Funct.

[39] Füstös J, Gramann K, Herbert BM, Pollatos O (2013). On the embodiment of emotion regulation: interoceptive awareness facilitates reappraisal. Soc Cogn Affect Neurosci.

[40] Brewer JA, Worhunsky PD, Gray JR, Tang YY, Weber J, Kober H (2011). Meditation experience is associated with differences in default mode network activity and connectivity. Proc Natl Acad Sci U S A.

[41] Hölzel BK, Carmody J, Evans KC, Hoge EA, Dusek JA, Morgan L, Pitman RK, Lazar SW (2010). Stress reduction correlates with structural changes in the amygdala. Soc Cogn Affect Neurosci.

[42] Segal Z, Williams M, Teasdale J (2013). Mindfulness-based cognitive therapy for depression.

[43] Williams JMG (2008). Mindfulness, depression and modes of mind. Cogn Ther Res.

[44] Mehling WE, Price C, Daubenmier JJ, Acree M, Bartmess E, Stewart A (2012). The multidimensional assessment of interoceptive awareness (MAIA). PLoS One.

[45] Cayoun BA, Sauvage V, Van Impe M (2004). A non diagnosis-specific application of mindfulness-based cognitive-behaviour therapy (MCBT): a pilot study.

[46] Sohrabi F, Sohrabi A, Shams-Alizadeh N (2014). Effects of mindfulness-integrated cognitive behaviour therapy (MiCBT) on depression, adherence and blood glucose control of patients with type 2 diabetes mellitus.

[47] Farzinrad B, Kamal MN. Comparison between Effectiveness of Mindfulness integrated Cognitive Behavioral Therapy (MiCBT) and Rational Emotional Behavior Therapy (REBT) on procrastination, perfectionism and worry in students. In: 6th International Congress on Child and Adolescent Psychiatry. East Azarbaijan: Tabtiz university of medical sciences; 2013.

[48] Bahrani S, Zargar F, Yousefipour G, Akbari H. The effectiveness of mindfulness-integrated cognitive behavior therapy on depression, anxiety, and stress in females with multiple sclerosis: a single blind randomized controlled trial. Iran Red Crescent Med J. 2017;19(4):p1–8.

[49] Yazdanimehr R, Omidi A, Sadat Z, Akbari H (2016). The effect of mindfulness-integrated cognitive behavior therapy on depression and anxiety among pregnant women: a randomized clinical trial. J Caring Sci.

[50] Cayoun BA, Simmons A, Shires A. Immediate and lasting chronic pain reduction following a brief self-implemented mindfulness-based interoceptive exposure task: a pilot study. Mindfulness. 2017:1–13.

[51] Scott-Hamilton J, Schutte NS, Brown RF (2016). Effects of a mindfulness intervention on sports-anxiety, pessimism, and flow in competitive cyclists. Appl Psychol Health Well Being.

[52] Francis SEB (2013). A preliminary study of mindfulness-integreatd cognitive behaviour therapy: results from a series of group interventions.

[53] Kessler RC, Andrews G, Colpe LJ, Hiripi E, Mroczek DK, Normand S-L, Walters EE, Zaslavsky AM (2002). Short screening scales to monitor population prevalences and trends in non-specific psychological distress. Psychol Med.

[54] Lovibond SH, Lovibond PF (1995). Manual for the depression anxiety stress scales.

[55] Henry JD, Crawford JR (2005). The 21-item version of the depression anxiety stress scales (DASS–21): normative data and psychometric evaluation in a large non-clinical sample. Br J Clin Psychol.

[56] Crawford JR, Henry JD (2003). The depression anxiety stress scales (DASS): normative data and latent structure in a large non-clinical sample. Br J Clin Psychol.

[57] Diener E, Emmons RA, Larsen RJ, Griffin S (1985). The satisfaction with life scale. J Pers Assess.

[58] Lawton MP (1975). The Philadelphia geriatric center morale scale: a revision. J Gerontol.

[59] Diener E, Wirtz D, Tov W, Kim-Prieto C, Choi D, Oishi S, Biswas-Diener R (2010). New well-being measures: short scales to assess flourishing and positive and negative feelings. Soc Indic Res.

[60] Lovibond PF, Lovibond SH (1995). The structure of negative emotional states: comparison of the depression anxiety stress scales (DASS) with the Beck depression and anxiety inventories. Behav Res Ther.

[61] Wallach H, Buchheld N, Buttenmuller V, Kleinknecht N, Schmidt S (2006). Measuring mindfulness. The Freiburg mindfulness inventory. Personal Individ Differ.

[62] Brown KW, Ryan RM (2003). The benefits of being present: mindfulness and its role in psychological well-being. J Pers Soc Psychol.

[63] Baer RA, Smith GT, Allen KB (2004). Assessment of mindfulness by self-report: the Kentucky inventory of mindfulness skills. Assessment.

[64] Armijo-Olivo S, Warren S, Magee D (2009). Intention to treat analysis, compliance, drop-outs and how to deal with missing data in clinical research: a review. Phys Ther Rev.

[65] Baer RA, Smith GT, Hopkins J, Krietemeyer J, Toney L (2006). Using self-report assessment methods to explore facets of mindfulness. Assessment.

[66] Kasselis N. A psychometric analysis of the mindfulness-based self efficacy scale (MSES): Honours Bachelor of Arts. Australia: University of Tasmania; 2011.

[67] Fresco DM, Moore MT, van Dulmen MH, Segal ZV, Ma SH, Teasdale JD, Williams JMG (2007). Initial psychometric properties of the experiences questionnaire: validation of a self-report measure of decentering. Behav Ther.

[68] Bernstein A, Hadash Y, Lichtash Y, Tanay G, Shepherd K, Fresco DM (2015). Decentering and related constructs:a critical review and metacognitive processes model. Perspect Psychol Sci.

[69] Wells A (2007). Cognition about cognition: metacognitive therapy and change in generalized anxiety disorder and social phobia. Cogn Behav Pract.

[70] Teasdale J, Moore R, Hayhurst H, Pope M, Williams S, Segal Z (2002). Metacognitive awareness and prevention of relapse in depression: empirical evidence. J Consult Clin Psychol.

[71] Soler J, Franquesa A, Feliu-Soler A, Cebolla A, García-Campayo J, Tejedor R, Demarzo M, Baños R, Pascual JC, Portella MJ (2014). Assessing decentering: validation, psychometric properties, and clinical usefulness of the experiences questionnaire in a Spanish sample. Behav Ther.

[72] Gueorguieva R, Krystal JH (2004). Move over anova: Progress in analyzing repeated-measures data andits reflection in papers published in the archives of general psychiatry. Arch Gen Psychiatry.

[73] Sahdra BK, Shaver PR, Brown KW (2010). A scale to measure nonattachment: a Buddhist complement to Western research on attachment and adaptive functioning. J Pers Assess.

[74] Lindahl JR, Fisher NE, Cooper DJ, Rosen RK, Britton WB (2017). The varieties of contemplative experience: a mixed-methods study of meditation-related challenges in Western Buddhists. PLoS One.

[75] Eisendrath SJ, Gillung EP, Delucchi KL, Chartier M, Mathalon DH, Sullivan JC, Segal ZV, Feldman MD (2014). Mindfulness-based cognitive therapy (MBCT) versus the health-enhancement program (HEP) for adults with treatment-resistant depression: a randomized control trial study protocol. BMC Complement Altern Med.

[76] Fritz MS, Mackinnon DP (2007). Required sample size to detect the mediated effect. Psychol Sci.

[77] Lau MA, Bishop SR, Segal ZV, Buis T, Anderson ND, Carlson L, Shapiro S, Carmody J (2006). The Toronto mindfulness scale: development and validation. J Clin Psychol.

[78] Turner L, Shamseer L, Altman DG, Weeks L, Peters J, Kober T, Dias S, Schulz KF, Plint AC, Moher D. Consolidated standards of reporting trials (CONSORT) and the completeness of reporting of randomised controlled trials (RTCs) published in mendical journals, reprint of a Cochrane review. Cochrane Libr. 2012;11:p1–10. 10.1002/14651858.MR000030.pub2 PMC7386818 23152285

[79] Sobel ME (1982). Asymptotoc confidence intervals for indirect effects in structural equations models. Sociol Methodol.

[80] Birnbaum HG, Kessler RC, Kelley D, Ben-Hamadi R, Joish VN, Greenberg PE (2010). Employer burden of mild, moderate, and severe major depressive disorder: mental health services utilization and costs, and work performance. Depress Anxiety.

