# Correction to: Study protocol for a randomized control trial to investigate the effectiveness of an 8-week mindfulness-integrated cognitive behavior therapy (MiCBT) transdiagnostic group intervention for primary care patients

**DOI:** 10.1186/s12888-020-02534-y

**Published:** 2020-03-26

**Authors:** Sarah Francis, Frances Shawyer, Bruno Cayoun, Joanne Enticott, Graham Meadows

**Affiliations:** 1 Southern Synergy, Department of Psychiatry, School of Clinical Sciences at Monash Health, Monash University, Clayton, Victoria 3800 Australia; 2 Department of Psychiatry, School of Clinical Sciences at Monash Health, Monash University, Dandenong Hospital, 126 - 128 Cleeland St, Dandenong, Victoria 3175 Australia; 3 Mindfulness-integrated Cognitive Behavior Therapy Institute, Hobart, Tasmania Australia; 4 Department of General Practice, School of Primary and Allied Health Care, Faculty of Medicine, Nursing and Health Sciences, Monash University, Melbourne, VIC Australia; 5 Mental Health Program, Monash Health, Melbourne, Victoria Australia; 6 Melbourne School of Population and Global Health, University of Melbourne, Parkville, Victoria 3010 Australia

**Correction to: BMC Psychiatry**

**https://doi.org/10.1186/s12888-019-2411-1**

After publication of our article [1] the authors have notified us that one of the names has been incorrectly spelled.

- Original name spelling:
  Sarah Frances
- Correct name spelling:
  Sarah Francis
